# CRISPR-Cas9-Mediated Intersectional Knockout of Glycogen Synthase Kinase 3β in D2 Receptor–Expressing Medial Prefrontal Cortex Neurons Reveals Contributions to Emotional Regulation

**DOI:** 10.1089/crispr.2019.0075

**Published:** 2020-06-17

**Authors:** Jivan Khlghatyan, Jean-Martin Beaulieu

**Affiliations:** ^1^ Department of Pharmacology and Toxicology, University of Toronto, Medical Sciences Building, Toronto, Canada; ^2^ Department of Psychiatry and Neuroscience, Faculty of Medicine, Université Laval, Québec-City, Canada.

## Abstract

Glycogen synthase kinase 3β (GSK3β) activity is regulated by dopamine D2 receptor signaling and can be inhibited by psychoactive drugs in a D2 receptor–dependent manner. However, GSK3β is ubiquitously expressed in the brain, and D2 receptor–expressing cells are distributed as a mosaic in multiple cortical regions. This complicates the interrogation of GSK3β functions in cortical D2 cells in a circuit-defined manner using conventional animal models. We used a CRISPR-Cas9-mediated intersectional approach to achieve targeted deletion of GSK3β in D2-expressing neurons of the adult medial prefrontal cortex (mPFC). Isolation and analysis of ribosome-associated RNA specifically from mPFC D2 neurons lacking GSK3β demonstrated large-scale translatome alterations. Deletion of GSK3β in mPFC D2 neurons revealed its contribution to anxiety-related, cognitive, and social behaviors. Our results underscore the viability of an intersectional knockout approach to study functions of a ubiquitous gene in a network-defined fashion while uncovering the contribution of GSK3β expressed in mPFC D2 neurons in the regulation of behavioral dimensions related to mood and emotions. This advances our understanding of GSK3β action at a brain circuit level and can potentially lead to the development of circuit selective therapeutics.

## Introduction

Gene products involved in disease processes and drug action are often expressed ubiquitously. Thus, their functional implication can be widely different in time (developmental stage) and space (brain region, neuronal circuit, and particular cell type). Traditional approaches have allowed systemic as well as region or cell type-selective gene knockouts. However, their resolution remains limited and does not allow for the investigation of gene functions within cellular subpopulations belonging to the same brain region or neuronal circuit at a specific developmental stage.

Glycogen synthase kinase 3β (GSK3β) is a serine-threonine kinase that is ubiquitously expressed across the brain during the entire life-span.^1,2^ This kinase regulates neurodevelopment,^2,3^ neuronal signaling, and plasticity.^4–7^ GSK3β is also involved in regulating mood,^8–11^ cognition,^12^ social interaction, and depressive-like behaviors.^8,12,13^ These functions of GSK3β have been identified by using drugs that can systemically modulate its activity or by knockout or knockdown approaches, including germline heterozygous knockout, knockout only in forebrain *CamK*II-expressing pyramidal neurons, Cre-mediated knockout in the adult prefrontal cortex (PFC), CRISPR-Cas9-mediated knockout only in adult medial PFC (mPFC) neurons, shRNA-mediated knockdown in adult nucleus accumbens shell neurons, and germline knockout in serotonergic neurons.^8,9,11,13–17^ However, the ubiquitous expression profile of GSK3β makes it difficult to pinpoint the neuroanatomical correlates of these various functions precisely.

Genome-wide association studies have revealed that genetic variants of the dopamine D2 receptor (D2) are associated with depression and schizophrenia.^18–20^ D2 is a G-protein-coupled receptor and a major target of antipsychotic drugs.^21^ This receptor has been shown to signal through Gαi/o to inhibit cAMP production and beta-arrestin-2 (βARR2) to inhibit AKT and activate GSK3β.^22–24^ Treatment with selective serotonin reuptake inhibitors (SSRIs) and ketamine also inhibit the activity of GSK3β.^16,25,26^ Furthermore, GSK3β activity can be inhibited by mood stabilizers in a D2- and βARR2-dependent manner.^9,15,27^ A study involving germline knockout of GSK3β in D2-expressing neurons indicated that GSK3β mediates the locomotor response to lithium and antipsychotics, most likely by interfering with D2 signaling in striatal medium spiny neurons.^28^ However, the potential contribution of GSK3β in the regulation of other behavioral dimensions by D2-expressing neurons of other brain regions remains unknown.

We recently identified multiple cortical regions with a mosaic distribution of D2-expressing neurons, ranging from 5% to 50% of total neurons within a given region.^29^ This raises questions about the role of GSK3β in D2-expressing neurons of these various cortical regions. However, it has been technically challenging to achieve knockout of GSK3β in a brain region–specific and cell type–specific manner at a given developmental stage. In other words, it has been impossible to eliminate GSK3β expression in a given brain region while targeting only D2 neurons. Considering the involvement of the mPFC in mental disorders and the presence of D2-expressing neurons in this region, knocking out GSK3β in adult mPFC D2 neurons represents an ideal model to explore approaches involving high-resolution gene targeting in a specific cellular subset belonging to brain region composed of a heterogeneous neuronal population.^18,29,30^

We used an intersectional approach involving a combination of single-guide RNA (sgRNA) viral delivery with Cre-mediated conditional Cas9 expression to target GSK3β only in mPFC D2 neurons of adult mice. The combination of this approach with a conditional RiboTag reporter system allowed ribosome-associated RNA to be extracted specifically from mPFC D2 neurons and demonstrated how GSK3β knockout affects the neuronal translatome. Moreover, knockout of GSK3β in mPFC D2 neurons uncovered its involvement in the regulation of anxiety-related, cognitive, and social behaviors.

## Methods

Note that detailed Methods are described in the Supplementary Data.

### Animals

D2Cre heterozygous bacterial artificial chromosome transgenic mice (GENSAT RRID: MMRRC_017263-UCD) were crossed to heterozygous Rosa26-LSL-Cas9 knockin mice (stock no.: 024857, The Jackson Laboratory, Bar Harbor, ME) to generate the D2Cre/LSL-Flag-Cas9 line. Male mice aged 2.5–3 months from this line were used for virus injections, immunohistochemistry, and behaviors.^31^

Homozygous knockin RiboTag mice were crossed to the D2Cre/LSL-Flag-Cas9 mouse line to generate D2Cre/LSL-Flag-Cas9/RiboTag mice.^32^ Male mice aged 2.5–3 months from this line were used for viral injections followed by ribosome-associated mRNA isolation and RNAseq.

Mice were maintained on a 12 h/12 h light/dark cycle with *ad libitum* access to food and water. All experiments conducted in this study were approved by the Université Laval and University of Toronto Institutional Animal Care Committee in line with guidelines from the Canadian Council on Animal Care.

### Mouse stereotaxic surgery and adeno-associated viruses

Mice were anesthetized with a preparation of 10 mg/mL ketamine and 1 mg/mL xylazine (0.1 mL/10g given intraperitoneally [i.p.]). The animal was placed in a stereotaxic frame, and the skull surface was exposed. Two holes were drilled at injection sites, and virus (AAV GFP or AAV Gsk3sgRNA/GFP) was injected using an injector with a microsyringe pump controller (World Precision Instruments, Inc., Sarasota, FL) at a speed of 4 nL/s. AAV GFP and AAV Gsk3sgRNA/GFP viral particles have been described previously.^11^ Following injection, coordinates for mPFC were used: anterior-posterior +2.4, medial-lateral ±0.5, and dorsal-ventral −1.7. All measures were taken before, during, and after surgery to minimize animal pain and discomfort.

### Immunohistochemistry and quantification

Mice were euthanized 3 weeks after viral delivery by a lethal dose of ketamine/xylazine and perfused with phosphate-buffered saline (PBS) followed by 4% paraformaldehyde (PFA). Brains were incubated in 4% PFA for 24 h at 4°C. Fixed tissue was sectioned using a vibratome (VT1000S; Leica, Wetzlar, Germany). Flag-Cas9 staining required antigen retrieval. Thus, 40 μm sections had to be boiled for 2 min in sodium citrate buffer (10 mM tri-sodium citrate dehydrate, 0.05% Tween-20, pH 6.0) and cooled at room temperature (RT) for 20 min. This resulted in the disappearance of endogenous GFP signal in D2Cre/LSL-Flag-Cas9 mice (see Supplementary Fig. 1C). However, virally expressed GFP signal was still present (Fig. 2). Sections were blocked and permeabilized with a permeabilization solution containing 10% normal goat serum and 0.5% Triton X-100 (Sigma–Aldrich, St. Louis, MO) in PBS for 2 h. Sections were incubated with primary antibodies diluted in permeabilization solution overnight at 4°C. After three washes in PBS, samples were incubated with secondary antibodies for 2 h at RT. After washing with PBS three times, sections were mounted using DAKO mounting medium (DAKO, Mississauga, Canada) and visualized with a confocal microscope (Zeiss LSM 880, Zen 2011 Software; Zeiss, Oberkochen, Germany).

For quantification, every third serial coronal section was taken, as shown in Supplementary Figure 1B. In every region of interest (e.g., prelimbic [PL] layer 5 or cingulate [Cg] layer 5) three randomly chosen Z-stack pictures were taken with a 60 × magnification objective. Quantification was performed manually using ImageJ (National Institutes of Health, Bethesda, MD).

The following primary antibodies were used: mouse anti-flag (1:500; F1804; Sigma–Aldrich), rabbit anti-Gsk3β (1:500; 9315; Cell Signaling Technology, Danvers, MA). The following secondary antibodies were used: goat anti-mouse Alexa 647 and goat anti-rabbit Alexa 568 (1:1,000; Invitrogen, Carlsbad, CA).

Cell-line culture, transfection, and Western blot have been described previously.^11^ Behavioral tests and Z-scoring was performed as described previously.^8,11,33–35^ Tissue dissection, immunoprecipitation of polyribosomes, and RNA isolation have been described previously.^29^

### Drugs

Methylphenidate (30 mg/kg) was dissolved in saline and given to mice i.p.

### Data analysis and statistics

Data are presented as means±standard error of the mean. Two-tailed *t*-tests were used in GraphPad Prism v5 (GraphPad Software, Inc., La Jolla, CA) for comparisons between two groups, with significance set at *p*-values of <0.05, <0.01, and <0.001.

## Results

### Efficient and specific knockout of GSK3β by CRISPR-Cas9

**FIG. 1.**
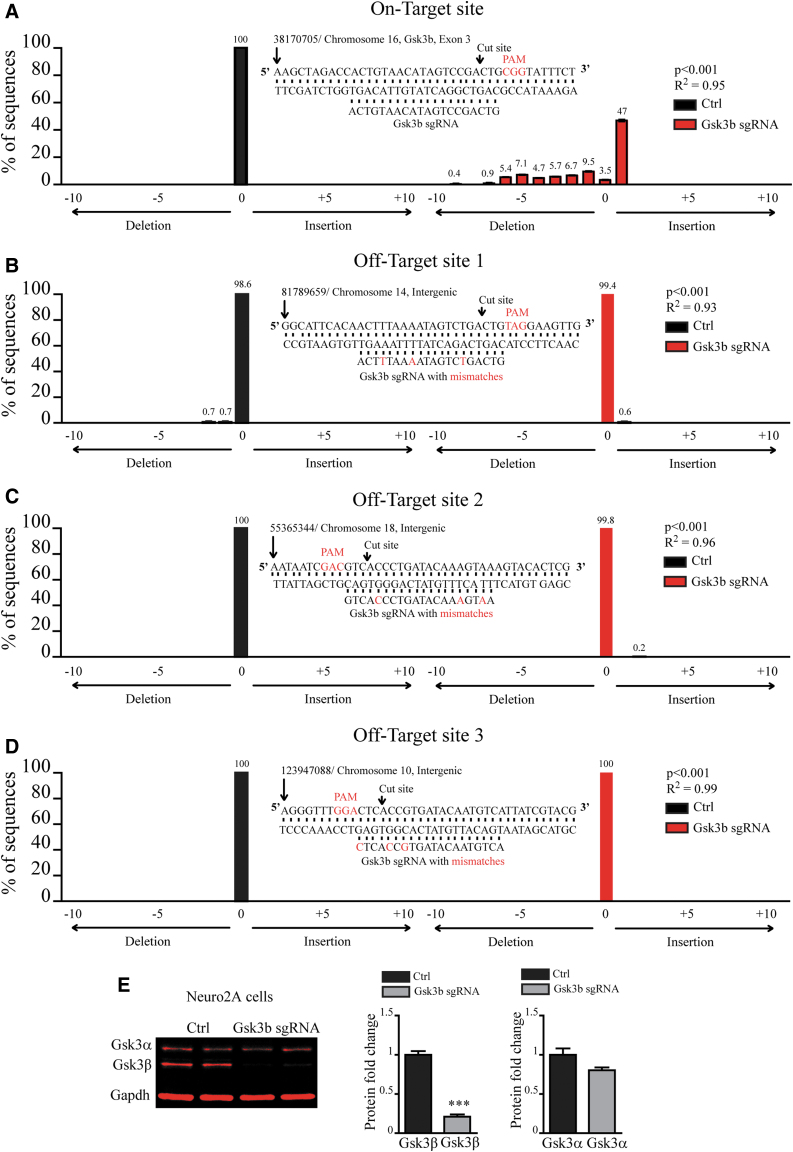
Specificity of CRISPR-Cas9-mediated Gsk3β knockout. Quantification of mutations using TIDE analysis at **(A)** on-target and **(B–D)** three putative off-target sites. Position “0” represents the percentage of non-mutated sequence, “–” positions are deletions, and “+” positions are insertions. Middle panels show the nucleotide sequence and chromosomal position at a putative target site, Gsk3b sgRNA, as well as mismatch nucleotides at off-target sites. **(E)** Quantification of the expression of Gsk3α and Gsk3β after CRISPR-Cas9-mediated knockout of Gsk3β in Neuro2A cells (Gsk3α: Ctrl 1 ± 0.08 *n* = 5, Gsk3b sgRNA 0.8 ± 0.035 *n* = 4; Gsk3β: Ctrl 1 ± 0.047 *n* = 5, Gsk3b sgRNA 0.21 ± 0.028 *n* = 5; *p < 0.05, Student's *t*-test). Left panel: an example of Western blot membrane stained for Gsk3α, Gsk3β, and Gapdh.

To investigate the efficacy and specificity of CRISPR-Cas9-mediated knockout of GSK3β, we tested the ability of previously characterized *Gsk3b* sgRNA to cut and induce mutations of DNA at on- and off-target sites.^11^ Targeting efficacy and specificity of the CRISPR-Cas9 system depends on the sequence of the guide RNA.^36,37^ It has been shown that genomic regions that contain few mismatches to the guide RNA sequence can represent potential off-target sites.^37^ We used two previously characterized algorithms to predict putative off-target sites.^37,38^ Both algorithms showed that the mouse genome does not contain sites with one or two mismatches to *Gsk3b* sgRNA. We identified only three putative off-target sites containing three mismatches (Fig. 1B–D). All three putative off-target sites were positioned in intergenic regions with no coding activity (Fig. 1B–D). Sites with four and more mismatches were disregarded, since predicted activity of sgRNA at those sites was virtually none.^36,37^ We transfected mouse Neuro2A cells with an “all in one” CRISPR plasmid containing previously characterized *Gsk3b* sgRNA or scrambled sgRNA as a control.^11^ Genomic DNA was isolated, and tracking of indels by decomposition (TIDE) analysis was applied to find activity at on-target (Gsk3b Exon 3) and off-target genomic loci (Fig. 1A–D).^39^ At the on-target site, in the Ctrl condition, no insertions or deletions were detected, and 100% of DNA sequences were not mutated (position “0”; Fig. 1A). At the on-target site, in the *Gsk3b* sgRNA condition, multiple deletions and insertions were detected, with one nucleotide insertion accounting for 47% of mutated DNA sequences (position “+1”; Fig. 1A). Overall, only 3.5% of DNA sequences were not mutated (position “0”; Fig. 1A). At all three off-target sites, virtually no mutations were detected in both Ctrl and *Gsk3b* sgRNA conditions (Fig. 1B–D).

Mutation in the exon of *Gsk3b* can disrupt its protein expression. Indeed, Western blot analysis showed a dramatic reduction of GSK3β expression in *Gsk3b* sgRNA transfected cells compared to controls, while GSK3α expression did not differ between conditions (Fig. 1E; data and statistics are indicated in the figure legends). Overall, this shows that CRISPR-Cas9-mediated knockout of GSK3β is robust and specific.

### Efficient knockout of GSK3β in mPFC D2 neurons of adult mice

To achieve somatic knockout of GSK3β exclusively in D2-expressing mPFC neurons (Gsk3sKO in mPFC D2), first, expression of Cas9 was activated only in D2 cells by crossing D2Cre mice with LSL-Flag-Cas9 mice.^31,40^ Second, *Gsk3b* sgRNA-containing virus (AAV Gsk3 sgRNA/GFP) or AAV GFP (as a control) were injected into the mPFC of these mice (Supplementary Fig. S1A).^11^ The selectivity of this D2Cre mouse line was demonstrated previously with Cre expression in PL layer 2/3 and layer 5, infralimbic (IL) layer 2/3 and layer 5, and Cg layer 5 of adult mouse mPFC.^29^

**FIG. 2.**
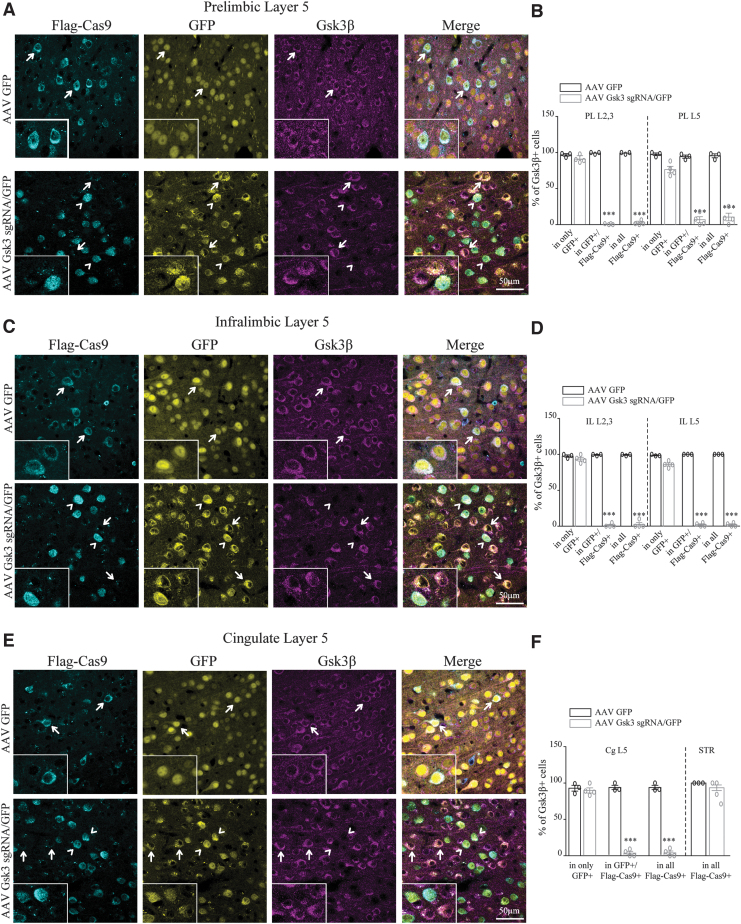
Knockout of GSK3β in D2 neurons of the medial prefrontal cortex (mPFC). **(A)** Immunofluorescent staining for Flag-Cas9 and Gsk3β in prelimbic (PL) layer 5 of virus-injected D2Cre/LSL-Flag-Cas9 mice. Insets show the zoomed picture. **(B)** Quantification of the percentage of Gsk3β-expressing cells (PL L2/3 AAV GFP condition: in only GFP 96.8 ± 1.7, in GFP+/Flag-Cas9 + 99.2 ± 0.75, in all Flag-Cas9 + 99.2 ± 0.75 *n* = 3. AAV Gsk3 sgRNA/GFP condition: in only GFP 90.9 ± 2.5, in GFP+/Flag-Cas9 + 0.6 ± 0.36, in all Flag-Cas9 + 2.2 ± 1.4 *n* = 4. PL L5 AAV GFP condition: in only GFP 97.5 ± 1.7, in GFP+/Flag-Cas9 + 94.3 ± 2.2, in all Flag-Cas9 + 95.5 ± 2.6 *n* = 3. AAV Gsk3 sgRNA/GFP condition: in only GFP 76 ± 5.4, in GFP+/Flag-Cas9 + 9 ± 4.1, in all Flag-Cas9 + 14 ± 4.8 *n* = 4; ***p < 0.0001, one-way analysis of variance [ANOVA]). **(C)** Immunofluorescent staining for Flag-Cas9 and Gsk3β in infralimbic (IL) layer 5 of virus-injected D2Cre/LSL-Flag-Cas9 mice. Insets show the zoomed picture. **(D)** Quantification of the percentage of Gsk3β-expressing cells (IL L2/3 AAV GFP condition: in only GFP 97.7 ± 1.6, in GFP+/Flag-Cas9 + 99.3 ± 0.6, in all Flag-Cas9 + 99.3 ± 0.6 *n* = 3. AAV Gsk3 sgRNA/GFP condition: in only GFP 92.2 ± 1.7, in GFP+/Flag-Cas9 + 1.6 ± 1.4, in all Flag-Cas9 + 3.4 ± 2.9 *n* = 4. IL L5 AAV GFP condition: in only GFP 98.6 ± 0.6, in GFP+/Flag-Cas9 + 100 ± 0, in all Flag-Cas9 + 100 ± 0 *n* = 3. AAV Gsk3 sgRNA/GFP condition: in only GFP 87.5 ± 1.5, in GFP+/Flag-Cas9 + 1.6 ± 1.4, in all Flag-Cas9 + 1.6 ± 1.4 *n* = 4; ***p < 0.0001, one-way ANOVA). **(E)** Immunofluorescent staining for Flag-Cas9 and Gsk3β in cingulate (Cg) layer 5 of virus-injected D2Cre/LSL-Flag-Cas9 mice. Insets show the zoomed picture. **(F)** Quantification of the percentage of Gsk3β-expressing cells (Cg L5 AAV GFP condition: in only GFP 92.9 ± 4, in GFP+/Flag-Cas9 + 93.9 ± 3, in all Flag-Cas9 + 93.9 ± 3 *n* = 3. AAV Gsk3 sgRNA/GFP condition: in only GFP 90.6 ± 4.2, in GFP+/Flag-Cas9 + 3.1 ± 2.6, in all Flag-Cas9 + 3.5 ± 3 *n* = 4. STR AAV GFP condition: in all Flag-Cas9 + 100 ± 0 *n* = 3. AAV Gsk3 sgRNA/GFP condition: in all Flag-Cas9 + 1.6 ± 1.4 *n* = 4; ***p < 0.0001, one way ANOVA). Error bars show standard error of the mean (SEM). Note that in the AAV GFP injected control condition, all cells express Gsk3β (indicated by arrows). In the AAV Gsk3 sgRNA/GFP injected condition, only cells that express Flag-Cas9 (corresponding to D2 cells) and sgRNA/GFP do not have Gsk3β signal (indicated by arrowheads), while cells having sgRNA/GFP but not Flag-Cas9 staining (not D2 cells) still express Gsk3β (indicated with arrows).

Three weeks after injection of AAV Gsk3 sgRNA/GFP or AAV GFP viruses, the infection area included PL, IL, and Cg cortexes, as shown by the expression of GFP in serial coronal sections of the mouse brain (Supplementary Fig. S1B).^11^ Thus, we investigated the ability of intersectional CRISPR-Cas9 to knock out GSK3β in D2 neurons of PL, IL, and Cg cortexes. Flag-Cas9 staining indicated the presence of Cas9, and virally expressed GFP signal indicated the presence of *Gsk3b* sgRNA (see Methods and Supplementary Fig. S1C). The vast majority of neurons containing Flag-Cas9 and *Gsk3* sgRNA (infected with AAV Gsk3 sgRNA/GFP) were devoid of GSK3β signal, while GSK3β staining was always present in neurons that only express GFP (infected with AAV Gsk3 sgRNA/GFP; Fig. 2A–F and Supplementary Fig. S1D and E). To determine the extent of the knockout, we also quantified the percentage of GSK3β-expressing Flag-Cas9 cells out of all Flag-Cas9 cells per brain region. Again, the vast majority of Flag-Cas9 cells were devoid of GSK3β staining, indicating widespread knockout (Fig. 2A–F and Supplementary Fig. S1D and E). Neurons infected with AAV GFP express GSK3β regardless of the presence of Flag-Cas9 (Fig. 2A–F and Supplementary Fig. S1D and E). Moreover, Flag-Cas9 neurons in the striatum of both AAV Gsk3 sgRNA/GFP– or AAV GFP–infected mice also express GSK3β (Fig. 2F and Supplementary Fig. S1F), since these neurons were not infected by viruses.

Overall, this demonstrates that we can achieve highly efficient somatic knockout of GSK3β exclusively in D2 neurons of the adult mPFC without affecting neighboring cells and D2 neurons in other brain regions.

### Gsk3sKO in mPFC D2 neurons alters the translatome

**FIG. 3.**
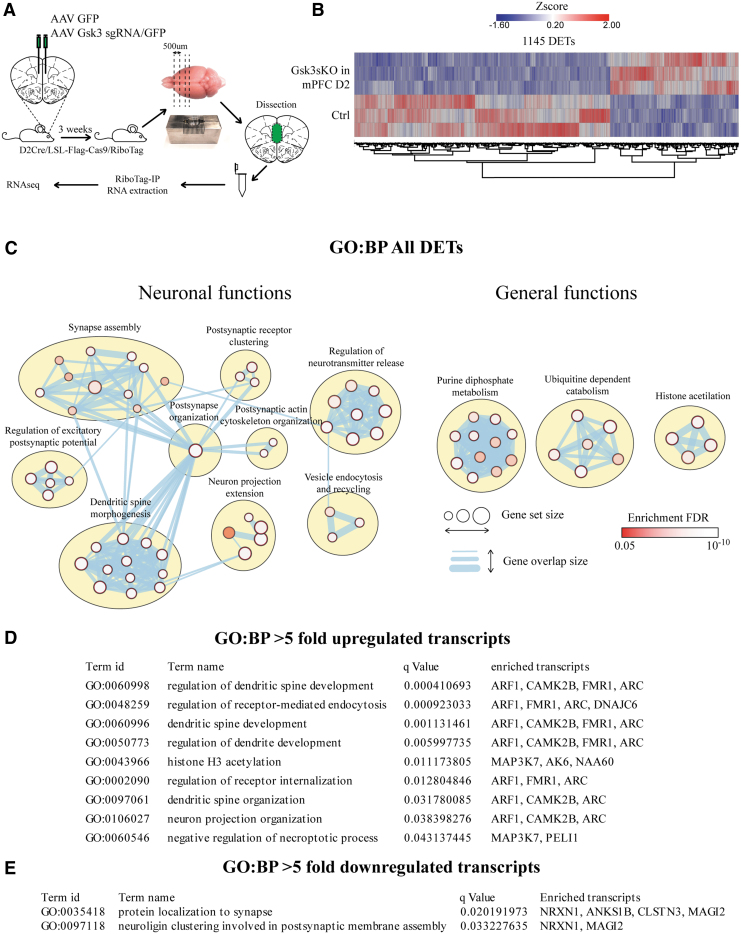
Knockout of GSK3β in D2 neurons of the mPFC induces translatome alterations. **(A)** Schematic representation of virus injection, tissue dissection, and RiboTag-IP RNA isolation. **(B)** Heat map summarizing differentially expressed transcripts (DETs) between Ctrl and Gsk3sKO in mPFC D2 conditions. **(C)** Enrichment of DETs in Gene Ontology Biological Processes (GO-BP). Visualization is made by cytoscape. **(D)** Enrichment of more than fivefold upregulated transcripts in GO-BP. **(E)** Enrichment of more than fivefold downregulated transcripts in GO-BP.

We used a Cre-activated RiboTag reporter system and RNAseq to investigate the impact of GSK3β knockout on the translatome of mPFC D2 cells. To isolate the translatome specifically from mPFC D2 neurons, we crossed D2Cre/LSL-Flag-Cas9 mice with RiboTag mice.^32^ D2Cre/LSL-Flag-Cas9/RiboTag mice expressed Cas9 and hemagglutinin-tagged Rpl22 ribosomal subunit only in D2 neurons. The D2Cre/RiboTag system has been shown to be robust and specific for the isolation of ribosome-bound RNA from D2 cells.^29^ AAV Gsk3sgRNA/GFP (Gsk3sKO in mPFC D2) or AAV GFP (Ctrl) was injected into the mPFC of D2Cre/LSL-Flag-Cas9/RiboTag mice. The mPFC was dissected 3 weeks after viral infection. RiboTag-immunoprecipitation and RNA extraction was performed, followed by RNAseq (Fig. 3A). We identified 1,145 unique differentially expressed transcripts (DETs) in Gsk3sKO in mPFC D2 compared to Ctrl (Fig. 3B and Supplementary Table S1). To identify biological dimensions based on DETs, we performed biological pathway enrichment (Gene Ontology Biological Processes [GO-BP]) using gProfiler and clustering using EnrichmentMap and ClusterMaker in Cytoscape (Fig. 3C, Supplementary Fig. S2, and Supplementary Table S2).^41^ We then manually separated enriched clusters into “neuronal” and “general” functions. The biggest and most enriched “neuronal function” clusters were related to postsynaptic structure and function as well as neurotransmission (Fig. 3C). The biggest and most enriched “general function” clusters were purine metabolism, ubiquitin-dependent catabolism, and histone acetylation. We then performed pathway enrichment analysis of more than fivefold upregulated (34 transcripts, Supplementary Table S1) or more than fivefold downregulated (81 transcripts, Supplementary Table S1) transcripts (Fig. 3D and E). In both cases, most of the enriched pathways were associated with synaptic functions (Fig. 3D and E).

Overall, this shows that intersectional CRISPR-Cas9-mediated manipulations can be combined with genome-wide translational readout. It also indicates that GSK3β knockout in mPFC D2 neurons can induce large-scale changes in the neuronal translatome, with the biggest effect on transcripts involved in synaptic functions.

### Gsk3sKO in mPFC D2 neurons exerts an anxiolytic-like effect on mouse behavior

**FIG. 4.**
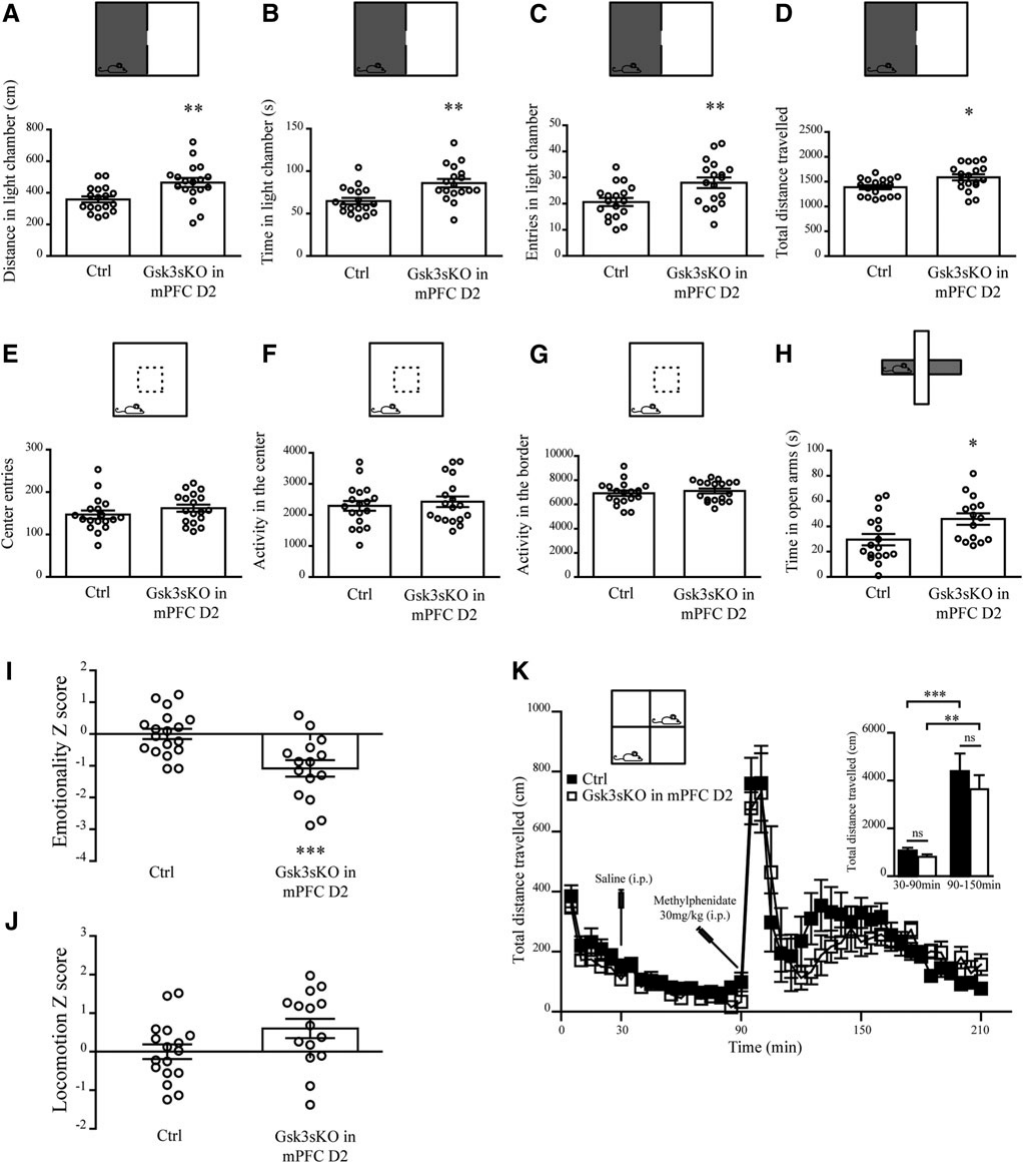
GSK3β of mPFC D2 neurons regulates anxiety-related behaviors. **(A–D)** Dark–light emergence test for Ctrl and Gsk3sKO in mPFC D2 mice. **(A)** Distance traveled in the light chamber (Ctrl: 357.9 ± 18.8 cm *n* = 18; Gsk3sKO in mPFC D2: 464.5 ± 29.1 cm *n* = 18). **(B)** Time spent in the light chamber (Ctrl: 64.6 ± 3.7 s *n* = 18; Gsk3sKO in mPFC D2: 85.9 ± 4.9 s *n* = 18). **(C)** Entries in the light chamber (Ctrl: 20.6 ± 1.5 *n* = 18; Gsk3sKO in mPFC D2: 28.00 ± 2.0 *n* = 18). **(D)** Total distance traveled (Ctrl: 1387 ± 41.3 cm *n* = 18; Gsk3sKO in mPFC D2: 1585 ± 61.6 cm *n* = 18). **(E–G)** Open field test for Ctrl and Gsk3sKO in mPFC D2 mice. **(E)** Center entries (Ctrl: 147 ± 9.5 *n* = 18; Gsk3sKO in mPFC D2: 162 ± 8.3 *n* = 18). **(F)** Activity in the center (Ctrl: 2293 ± 159.1 *n* = 18, Gsk3sKO in mPFC D2: 2425 ± 170.1 *n* = 18). **(G)** Activity in the border (Ctrl: 6923 ± 227 *n* = 18, Gsk3sKO in mPFC D2: 7111 ± 198 *n* = 18). **(H)** Elevated plus maze test for Ctrl and Gsk3sKO in mPFC D2 mice. Time in open arms (Ctrl: 29.5 ± 4.5 s *n* = 17; Gsk3sKO in mPFC D2: 45.8 ± 4.5 s *n* = 15). **(I)** Emotionality *Z*-score (Ctrl: −0.0000000033 ± 0.16 *n* = 18; Gsk3sKO in mPFC D2: −1 ± 0.25 *n* = 15). **(J)** Locomotion *Z*-score (Ctrl: −0.00000001 ± 0.19 *n* = 18; Gsk3sKO in mPFC D2: 0.6 ± 0.25 *n* = 15). **(K)** Total distance traveled every 5 min for Ctrl and Gsk3sKO in mPFC D2 mice after injection of saline at 30 min and methylphenidate at 90 min. Insert on the right shows total distance traveled between 30 and 90 min (Ctrl 1067 ± 121.2 *n* = 11; Gsk3sKO in mPFC D2 805.4 ± 105.8 *n* = 10) and 90 and 150 min (Ctrl 4384 ± 750.0 *n* = 11; Gsk3sKO in mPFC D2 3639 ± 590.8 *n* = 10). Error bars show SEM. *p < 0.05; **p < 0.01; ***p < 0.001; Student's *t*-test.

Behavioral consequences of GSK3β expression in adult D2-expressing mPFC neurons were then investigated. First, Ctrl and Gsk3sKO in mPFC D2 mice were subjected to several anxiety-related behavioral tests. In the dark–light emergence test, Gsk3sKO in mPFC D2 mice spent more time, traveled longer distance, and performed more entries in the light chamber compared to control (Fig. 4A–D). The open field test did not reveal significant differences between the two groups (Fig. 4E–G), and in the elevated plus maze test, Gsk3sKO in mPFC D2 mice spent more time in the open arms compared to Ctrl mice (Fig. 4H). To obtain a more complete understanding of anxiety status, we summarized the results across all the tests by performing behavioral Z-scoring for all mice.^33,35^ Mice from Gsk3sKO in the mPFC D2 group showed a decrease in the emotionality Z-score compared to control mice (Fig. 4I), while their locomotion Z-score was unaffected (Fig. 4J).

To evaluate whether GSK3β in mPFC D2 can affect hyperactivity, Ctrl and Gsk3sKO in mPFC D2 mice were treated with the dopamine reuptake inhibitor methylphenidate (30 mg/kg i.p.), and locomotion was measured (Fig. 4K) before and after drug administration. Mice from both groups showed no difference in exploratory locomotor activity prior to drug administration. Furthermore, in both groups, drug-induced hyperlocomotion was not affected by the knockout of GSK3β in mPFC D2 (Fig. 4K).

This indicates that a reduction of GSK3β expression in D2 neurons of the mPFC is sufficient to impact anxiety-related behaviors and does not affect locomotor behaviors.

### Gsk3β in mPFC D2 neurons is implicated in cognitive and social behaviors

**FIG. 5.**
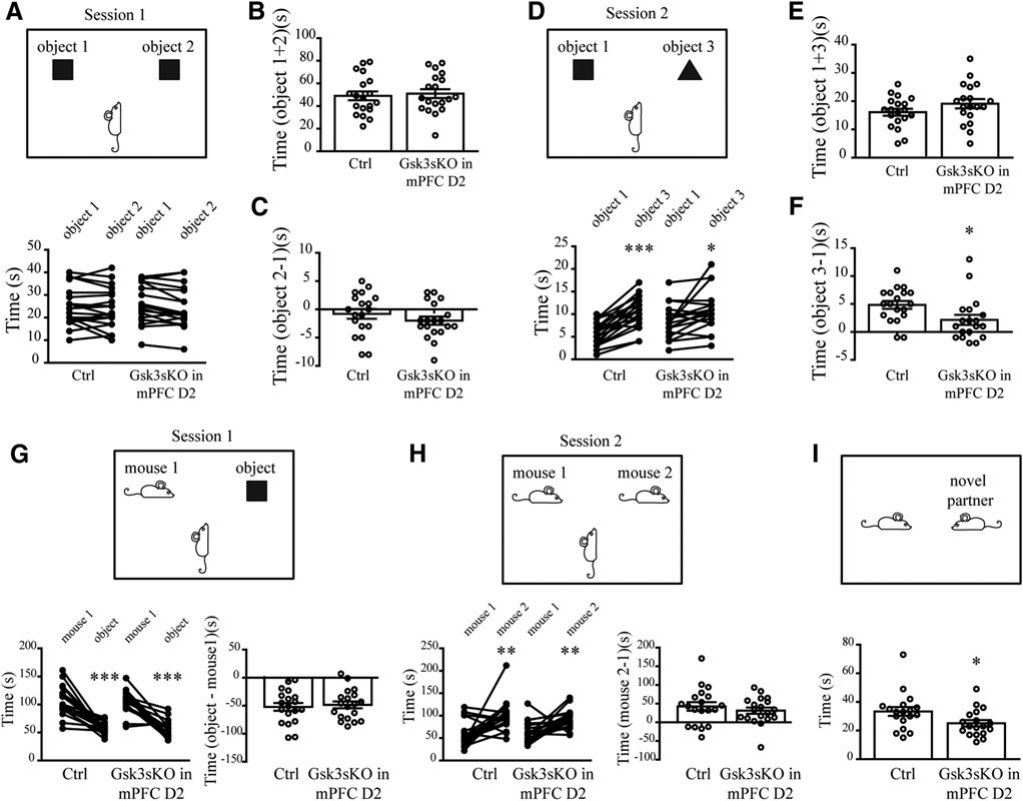
GSK3β of mPFC D2 neurons contributes to cognitive and social behaviors. **(A)** Novel object recognition (NOR) test for Ctrl and Gsk3sKO in mPFC D2 mice in session 1. **(B)** Total time spent with both objects (session 1 Ctrl: 49 ± 3.9 s *n* = 19; Gsk3sKO in mPFC D2: 50.9 ± 3.8 s, *n* = 19). **(C)** Difference in time spent with object 2 versus object 1 (session 1 Ctrl: −0.7 ± 0.8 s *n* = 19; Gsk3sKO in mPFC D2: −2 ± 0.7 s *n* = 19). **(D)** NOR test for Ctrl and Gsk3sKO in mPFC D2 mice in session 2 (*p < 0.05; ***p < 0.001; paired *t*-test). **(E)** Total time spent with both objects (session 2 Ctrl: 16.1 ± 1.2 s *n* = 19; Gsk3sKO in mPFC D2: 19.1 ± 1.6 s *n* = 19). **(F)** Difference in time spent with object 3 versus object 1 (session 2 Ctrl: 4.8 ± 0.7 s *n* = 19; Gsk3sKO in mPFC D2: 2.1 ± 0.9 s *n* = 19; *p < 0.05; Student's *t*-test). Preference for social novelty for Ctrl and Gsk3sKO in mPFC D2 mice. **(G)** Session 1 (time difference object–mouse: Ctrl: −52 ± 6.8 s *n* = 19; Gsk3sKO in mPFC D2: −48.4 ± 6.1 s *n* = 19). **(H)** Session 2 (time difference mouse 2–mouse 1: Ctrl: 42.5 ± 11.5 s *n* = 19; Gsk3sKO in mPFC D2: 31.3 ± 8.3 s *n* = 19; **p < 0.01; ***p < 0.001; paired *t*-test). **(I)** Free social interaction test for Ctrl and Gsk3sKO in mPFC D2 mice. Time in interaction (Ctrl: 33.3 ± 3.1 s *n* = 18; Gsk3sKO in mPFC D2: 25.1 ± 2.2 s *n* = 18; *p < 0.05; Student's *t*-test). Error bars show SEM.

Cortical GSK3β has been shown to be involved in cognitive and social behaviors.^8,12^ Thus, we expanded behavioral testing of both groups of mice to assess working memory and social interactions. In the novel object recognition (NOR) test, Gsk3sKO in mPFC D2 mice showed less robust discrimination between novel and familiar objects (Fig. 5A–F). This is indicative of mild short-term memory impairments.

In the three-chamber social test, Gsk3sKO in mPFC D2 mice did not differ from control mice in their preference for social novelty (Fig. 5G and H). However, mice in Gsk3sKO in mPFC D2 group spent less time in free social interaction compared to controls (Fig. 5I).

Overall, this suggests that GSK3β in mPFC D2 neurons may contribute to short-term memory and social behaviors.

## Discussion

The results presented here underscore the involvement of GSK3β in cortical D2 neurons in different behavioral modalities and demonstrate the usefulness of CRISPR-Cas9-mediated intersectional knockout strategies to investigate the functions of ubiquitously expressed genes in complex tissues.

D2 receptors are expressed in a mosaic of cell populations across different cortical regions.^29^ GSK3β is ubiquitously present throughout the brain and is regulating a variety of processes such as neuronal development and synaptic plasticity.^2,3,5,6^ Among these functions, GSK3β is an important mediator of D2 signaling that can be involved in the effects of lithium and antipsychotic drugs.^24,27,28^ D2 receptor signaling in different brain regions is believed to contribute to different behavioral dimensions under basal conditions and in response to drug treatment.^42^ Thus, it is required to target GSK3β gene expression only in D2 cells of a given brain region during adulthood if one wants to bypass developmental effects and understand the circuit correlate of its involvement in behavioral regulation. However, this type of genetic manipulation has been technically challenging. We overcame this limitation by combining AAV-mediated delivery of *Gsk3b* sgRNA in mice expressing Cas9 only in D2 neurons and achieved efficient and specific high-resolution knockout of GSK3β only in D2 neurons of the adult mouse mPFC (Fig. 2). One recent study also used an intersectional CRISPR-Cas9-mediated knockout to delete dopamine beta hydroxylase from a defined cluster of tyrosine hydroxylase–expressing neurons in the locus coeruleus to decouple the effects of the release of norepinephrine from changes in neuronal activity.^43^ Along with our results, this shows that intersectional CRISPR-Cas9 techniques can have broad applications.

We investigated whether GSK3β of mPFC D2 neurons is involved in the regulation of anxiety-related, cognitive, and social behaviors. These behaviors are known to be affected when manipulating GSK3β activity in a less selective manner.^44^

Knockout of GSK3β in mPFC D2 neurons resulted in anxiolytic-like behaviors (Fig. 4). In line with our findings, we previously showed that CRISPR-Cas9-mediated somatic knockout of GSK3β in all neurons of the mPFC also results in anxiolytic-like behaviors.^11^ Moreover, decrease anxiety-related behaviors were documented in forebrain pyramidal neuron GSK3β knockout mice and adult PFC GSK3β knockout mice.^8,9^ However, knockout of GSK3β in all D2 neurons of the brain failed to show anxiety-related effects.^28^ These differences can be explained by a lack of brain region selectivity and potential developmental effects of deleting GSK3β in all neurons of the brain that expressed D2 either at the adult stage or during development, which can alter the proper formation of neuronal networks. Stimulation of D2 receptor results in an increase of GSK3β activity.^44^ Interestingly, elevated D2 receptor availability in the PFC was found in patients with anxiety disorder compared to healthy subjects.^45^ Moreover, a decrease in anxiety symptoms after therapy was significantly correlated with the decrease of D2 receptor availability in the PFC.^46^ These findings strongly support a role of GSK3β in the regulation of anxiety-related behaviors downstream of D2 receptors in the mPFC of the adult brain.

Knockout of GSK3β in mPFC D2 neurons negatively impacted working memory and free social interaction (Fig. 5). Previous reports concerning the involvement of brain GSK3β in the regulation of these behavioral dimensions have been contradictory. In contrast to our findings, gain of function resulting from germline transgenic mice expressing constitutively active GSK3β (Gsk3β knockin mice) show impairments in NOR.^12^ In addition, heterozygous germline GSK3β knockout mice show no difference in social interactions, while forebrain pyramidal neuron GSK3β knockout mice show prosocial effects compared to controls in a free interaction test.^8^ Differences between these models can once again arise from developmental effects and circuit-specific involvement of GSK3β. In line with our observations, antagonism of D2 receptors in the PFC of adult rats impaired NOR and social novelty discrimination.^47^ This highlights that the involvement of GSK3β in the regulation of cognitive and social behaviors is cell type, brain region, and developmental stage selective.

The effects of GSK3β knockout on behaviors can be mediated by its action on synaptic transmission and plasticity. We have recently shown that knockout of GSK3β results in an increase in fragile X mental retardation syndrome-related protein 1 (FXR1).^9,11^ The regulation of FXR1 by GSK3β involves a post-translational mechanism involving the phosphorylation of FXR1 by the kinase. CRISPR-Cas9-mediated knockout of GSK3β in neuronal cultures affected AMPA receptors, and this effect was mediated via FXR1.^48^ CRISPR-Cas9-mediated somatic knockout of GSK3β or an increase in FXR1 expression in the adult mouse mPFC causes a decrease in AMPA-mediated spontaneous currents and a reduction in anxiety-like behaviors.^11^ This suggests that GSK3β–FXR1 interaction is important in the regulation of AMPA receptors and anxiety-related behaviors. However, further investigations are needed to elucidate the role of GSK3β on neurotransmission and plasticity specifically in D2-expressing neurons of the mPFC.

In addition to behavioral changes, the combination of CRISPR-Cas9 knockout with the RiboTag reporter system allowed ribosome-associated RNA to be extracted specifically from mPFC D2 neurons, and showed that GSK3β knockout has a large impact on the neuronal translatome (Fig. 3). Particularly, transcripts affected by GSK3β knockout were enriched in synaptic structure and function, neurotransmission, purine metabolism, ubiquitin-dependent catabolism, and histone acetylation, among others. Pathway enrichment analysis of highly affected (more than fivefold up- or downregulated) transcripts indicated that GSK3β knockout largely impacts synaptic function-related pathways (Fig. 3). In addition to previously discussed findings, these results indicate that the effect of GSK3β on behaviors can be mediated by its impact on synaptic functions in the D2 cells of the mPFC. Overall, this underscores the possibility of obtaining a translatome footprint of a given gene knockout in a brain region and cell type-selective manner.

Inhibition of GSK3β activity has been reported to occur in response to SSRIs, ketamine, antipsychotics, some anticonvulsants (mood stabilizers), and lithium.^9,15,16,25–27^ Furthermore, data from animal models support a role for GSK3β activity in the effect of these drugs.^44^ It is probable that subsets of these effects are explainable by the modulation of GSK3β downstream of D2 receptors in different brain regions. Our results indicate that GSK3β in adult mPFC neurons expressing D2 contributes to cognitive, social, and emotional dimensions of behavioral regulation. This regulation can for example contribute to the regulation of mood by lithium or of negative symptoms by antipsychotics in schizophrenia.

## Conclusion

Intersectional approaches have proven important to the precision of functional studies of neuronal circuits using optogenetics. Our results also underscore the viability of this approach to study gene functions in a network-defined fashion. This can advance our understanding of drug action at the brain circuit level and potentially lead to the development of circuit selective therapeutics.

## Supplementary Material

Supplemental data
