# Supplementary material for: CRISPR-Cas9-Mediated Intersectional Knockout of Glycogen Synthase Kinase 3β in D2 Receptor–Expressing Medial Prefrontal Cortex Neurons Reveals Contributions to Emotional Regulation
Source: CRISPR J. 2020 Jun 17;3(3):198–210. doi: 10.1089/crispr.2019.0075 (PMC7307679; doi:10.1089/crispr.2019.0075)
Supplement: Supplemental data [file Supp_Data.pdf]

Supplementary Data

Supplementary Methods

Tissue dissection, immunoprecipitation of polyribosomes, and RNA isolation

Mice were killed by rapid cervical dislocation. The heads of the animals were immediately cooled by immersion in liquid nitrogen for 6 s. First, serial coronal sections 500 μm thick were prepared using an ice-cold adult mouse brain slicer and matrix (Zivic Instruments, Pittsburgh, PA). Second, mPFC was dissected on an ice-cold surface using a microsurgical knife (KF Technology, Rome, Italy). Immunoprecipitation of polyribosomes was performed as described before.[S1] Tissue samples were lysed in homogenization buffer (50 mM Tris, pH 7.5, 100 mM KCl, 12 mM $MgCl_2$, 1% Nonidet P-40, 1 mM DTT, 100 IU/mL RNase Out, 100 μg/mL cycloheximide, Sigma protease inhibitor mixture) followed by centrifugation for 10 min at 10,000 *g*. Anti-hemagglutinin antibody (1:150; MMS-101R; BioLegend, San Diego, CA) was added to the collected supernatant, and the tubes were kept under constant rotation for 4 h at 4°C. Protein G magnetic beads (Life Technologies, Carlsbad, CA) were washed three times with homogenization buffer, added to the mixture, and kept on constant rotation overnight at 4°C. The following day, magnetic beads were washed three times with high salt buffer (50 mM Tris, pH 7.5, 300 mM KCl, 12 mM $MgCl_2$, 1% Nonidet P-40, 1 mM DTT, 100 IU/mL RNase Out, 100 μg/mL cycloheximide, Sigma protease inhibitor mixture). RNA was extracted by adding TRI reagent (Zymo Research, Irvine, CA) to magnetic beads pellet followed by Direct-zol RNA kit according to the manufacturer's instructions (Zymo Research). The RNA concentration was quantified using ND-1000 Spectrophotometer (NanoDrop Technologies, Wilmington, DE).

For RNAseq analysis, RNA was isolated from nine mice per group. Each group consisted of three biological replicates, and each replicate represented RNA pooled from three mice.

RNAseq analysis

Quality control. The quality control metrics for the RNAseq data were obtained using the tool RNA-SeQC (v1.1.7; for more information, see www.broadinstitute.org/cancer/cga/rna-seqc). This program takes aligned files as input and delivers a series of plots and statistics for each sample. Based on the output for each sample, the RNA sequencing quality was deemed acceptable for further analysis.

Processing pipeline. All raw FASTQ files were aligned to the appropriate mouse genome (GRCm38) using the HISAT2 aligner. HISAT2 is a fast and sensitive alignment program that uses a large set of small graph FM (GFM) indexes that collectively cover the whole reference genome. These local indexes, in conjunction with a series of alignment strategies, ensure rapid and accurate alignment of sequencing reads. Accessory programs for the alignment stage include SAMTOOLS (v1.3.1) and BEDTOOLS (v2.26.0). Alignment files were sorted by their genomic location and indexed using SAMTOOLS. These sorted binary SAM (BAM) files were then used as input for StringTie (v1.3.4), which assembles RNAseq alignments into potential transcripts. It uses a novel network flow algorithm, as well as an optional *de novo* assembly step to assemble and quantitate full-length transcripts representing multiple splice variants for each gene locus. Finally, in order to identify differentially expressed genes between samples, the Ballgown R-package was implemented (v3.4.3). Transcript-level FPKMs were estimated using Tablemaker. Expression was estimated for each transcript, exon, and intron (junction) in the assembly. All of the statistical analysis (organization, visualization, etc.) was conducted with the tools available within the Ballgown package.

Differential expression analysis. The statistical test applied to this data was a parametric *F*-test comparing nested linear models; details are available in the Ballgown manuscript. Briefly, two models are fit to each feature using the expression as the outcome: one including the covariate of interest (e.g., case/control status), and one not including that covariate. An *F*-statistic and *p*-value are calculated using the fits of the two models. A significant *p*-value means that the model including the covariate of interest fits significantly better than the model without that covariate, indicating differential expression. All the DETs with a *p*-value of <0.05 were selected for further analysis. Differential expression testing was carried out for Ctrl versus Gsk3sKO in D2 comparison. A heat map of gene expression *Z*-score (Fig. 3) was generated using the Morpheus tool (https://software.broadinstitute.org/morpheus/). The *Z*-score for gene expression was calculated using the formula $Z=(X-\mu)/\sigma$, which indicates how many standard deviations (σ) the level of expression of a given gene (X) is above or below the average expression from all the samples (μ).

Gene set enrichment analysis. First, DETs were filtered. All selected transcripts had a mean expression of

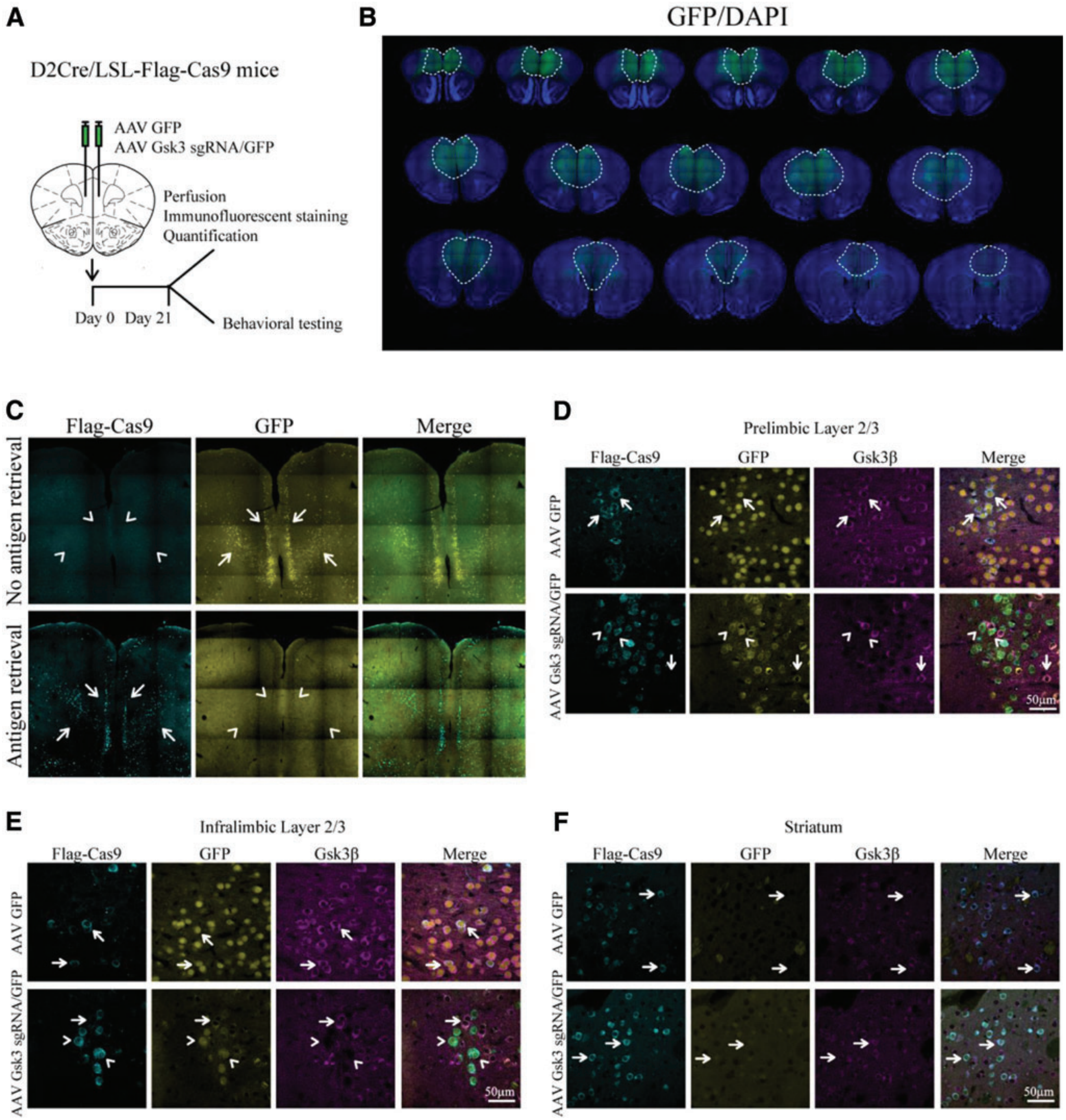

SUPPLEMENTARY FIG. S1. Cas9 expression and knockout of Gsk3β in mPFC D2 neurons. **(A)** Schematic representation of stereotaxic injection of viruses and experimental design. **(B)** Serial coronal sections showing the infection volume of the AAV GFP and AAV Gsk3 sgRNA/GFP viruses. **(C)** Flag-Cas9 staining is detected after antigen retrieval in mPFC. Endogenous GFP signal in D2Cre/LSL-Flag-Cas9 mice disappears after antigen retrieval. Arrows show presence and arrowheads show absence of fluorescent signal. **(D–F)** Immunofluorescent staining for Flag-Cas9 and Gsk3β in **(D)** prelimbic layer 2/3, **(E)** infralimbic layer 2/3. **(F)** Striatum of virus injected D2Cre/LSL-Flag-Cas9 mice. Note that in the AAV GFP–injected control condition, all cells express Gsk3β (indicated by arrows). In AAV Gsk3 sgRNA/GFP–injected condition, only cells that express Flag-Cas9 (corresponding to D2 cells) and sgRNA/GFP do not have Gsk3β signal (indicated by arrowheads), while cells having sgRNA/GFP but not Flag-Cas9 staining (not D2 cells) still express Gsk3β (indicated with arrows). In the striatum, all cells in both conditions express Gsk3β, since the viruses were injected into mPFC and did not infect the striatum.

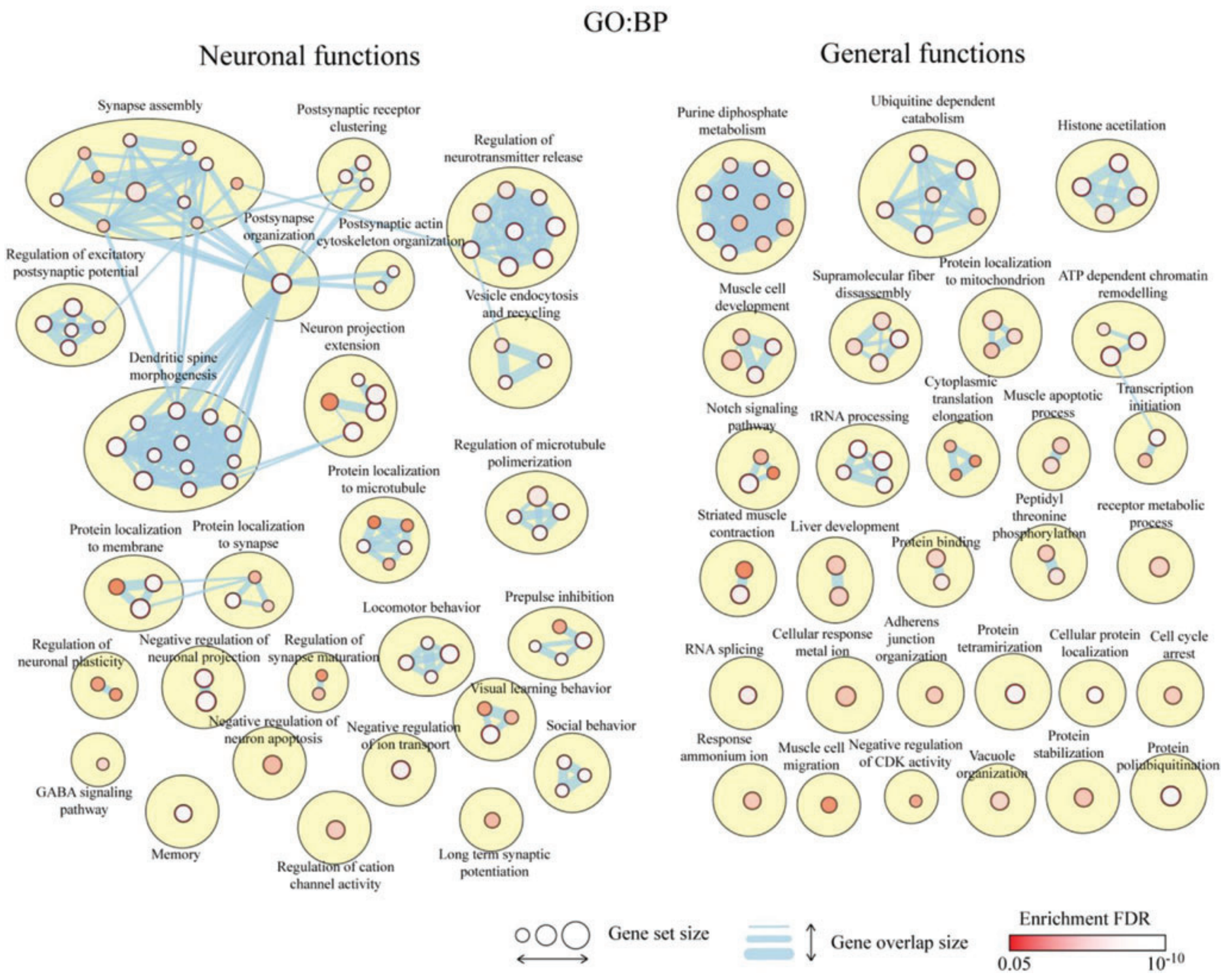

SUPPLEMENTARY FIG. S2. Enrichment of DETs in biological pathways. Enrichment of DETs between Ctrl and Gsk3sKO in mPFC D2 in GO-BP. Visualization is made by cytoscape.

>0.5 FKPM. The fold change (FC) threshold was set to 0.7–1.3. Enrichment analyses and visualization were performed following the pipeline described in Reimand *et al*.[S2] Enrichment analysis was performed using gProfiler (https://biit.cs.ut.ee/gprofiler/gost). Term size for pathways was selected to be min = 5 and max = 150. Only pathways passing significance threshold of 0.05 were selected. Gene enrichment in Gene Ontology Biological Processes (GO-BP) were selected. Then .GEM and .GMT files were downloaded from gProfiler and were used in the EnrichmentMap app of Cytoscape (v3.7.1) for visualization. The following parameters of EnrichmentMap were used: FDR q-value cutoff <0.05, Jaccard combined >0.375, overlap >0.5, and Prefuse Force Directed layout was chosen. Then, the ClusterMaker2 app was used to cluster enriched pathways based on similarity, and the AutoAnnotate and WordCloud apps were used to name clusters of enriched pathways using default parameters. The final pictures of the clusters of enriched pathways are shown in Figure 3 and Supplementary Figure S2. The list of all the enriched pathways is shown in Supplementary Table S2.

Cell line culture, transfection, Western blot, and TIDE analysis

Neuro-2A cells were grown in high glucose Dulbecco's modified Eagle's medium containing 10% fetal bovine serum, penicillin/streptomycin, and L-glutamine (HyClone;

GE Healthcare, Logan, UT). Cells were maintained at 37°C in 5% CO_2 atmosphere and transfected using Lipofectamine 2000 (Thermo Fisher Scientific, Waltham, MA) according to the manufacturer's protocols.

For TIDE analysis and *in vitro* evaluation of Gsk3β and Gsk3α expression by Western blot (Fig. 1), 50–70% confluent N2A cells were transfected with previously validated all-in-one px459-based constructs (pX459 vectors with guide targeting Gsk3b).[S3] To select only transfected cells, 48 h after transfection, cells were incubated with 3 μM puromycin for 72 h followed by 48 h of incubation without puromycin.

For Western blot, cells were washed and lysed on day 7 in lysis buffer containing: 50 mM Tris-HCl, 150 mM NaCl, 5 mM EDTA, Protease inhibitor cocktail (Sigma–Aldrich; P8340; 1×), 1% sodium dodecyl sulfate, 0.5% sodium deoxycholate, 1% NP-40, 10 mM sodium fluoride, 25 mM β-glycerophosphate, 10 mM sodium orthovanadate (Sigma–Aldrich, Oakville, Canada). Lysates were centrifuged at 10,000 *g* for 30 min, and supernatants were collected. Protein concentration was measured by using a DC-protein assay (Bio-Rad, Hercules, CA). Protein extracts were separated on precast 4–20% Tris-glycine gels (Thermo Fisher Scientific, Waltham, MA) and transferred to nitrocellulose membranes. Blots were immunostained overnight at 4°C with primary antibodies. Immune complexes were revealed using appropriate IR dye-labeled secondary antibodies from Li-Cor Biotechnology (Lincoln, NE). Quantitative analyses of fluorescent IR dye signal were carried out using Odyssey Imager and Image Studio Lite v5.2 (Licor Biotechnology, Lincoln, NE). For quantification, GAPDH was used as a loading control for the evaluation of total protein levels. The following primary antibodies were used in the experiments: mouse anti-GAPDH (1:5,000, Santa Cruz; sc-322333), mouse anti-Gsk3α/β (1:500; Santa Cruz; sc-7291). The following secondary antibodies were used: goat anti-mouse IR Dye 680 (1:10,000, Mandel; 926-68020).

To isolate genomic DNA for TIDE analysis,[S4] cells were lysed by tail buffer (0.1 M Tris, pH = 8.0, 0.2 M NaCl, 5 mM EDTA, 0.4% SDS, and 0.2 mg/mL proteinase K), and DNA was precipitated using isopropanol followed by centrifugation (13,000 *g* for 15 min). DNA was re-suspended in TE Buffer (10 mM Tris, pH 8.0, 0.1 mM EDTA) and used for PCR. The following primers were used to amplify the on-target site: forward GGTTCC TCTTGCCCCCTATTA; reverse TTCTCATTGGCAT TTCCACGC. The following primers were used to amplify the three putative off-target sites: site 1: forward TCATTATAGGTCTCGGGGCAAG, reverse AAATG ATGAAGGAATTTGGTCGGAA; site 2, forward CC TGCTGTCTCTCCCTTGTG, reverse GGAAGCCTAC TGCAAGAGCA; site 3, forward AACGTGAAACTT TGTTTGCAATATC, reverse ACAAATTTCAATC TGTGGCTGGG. PCR products were sent to sequencing with forward primers, and frequencies of mutations were determined by online TIDE tool (https://tide.nki.nl/).[S4]

Behavioral tests

Open field test. The open field test (OFT) was performed for 30 min in an automated Omnitech Digiscan apparatus (AccuScan Instrument, Columbus, OH). Each mouse was placed in a corner of a large Plexiglas box, and the exploratory activity was recorded. A number of entries, time, horizontal activity, and total distance were recorded separately for the central (25% of the total surface) and peripheral areas.

Dark–light emergence test. The dark–light emergence test (DLET) was performed for 5 min with mice placed initially at the center of the dark chamber. Tests were conducted using an automated open field activity apparatus with light/dark insert (Med-Associates, St Albans, VE) with the light compartment illuminated at 800 lux. The total time spent in the dark and light compartments, the total distance traveled, and the number of entries from the dark to the light chamber were used as parameters for analysis.

Elevated plus maze. The elevated plus maze (EPM) was performed for 5 min, with mice initially placed in the far end of the close arm. Mice were video tracked using Viewer software (Biobserve Behavioral Research). The time spent in the open arm was measured and used for the analysis.

Behavioral Z-scoring. To obtain integrated measures in each group, emotionality- and locomotion-related data were normalized using a *Z*-score methodology.[S5] *Z*-scores for individual animals were calculated using the formula $Z = (X - \mu)/\sigma$, which indicates how many standard deviations (σ) an observation (X) is above or below the mean of a control group (μ). *Z*-scores for behavioral measures were averaged first within the test and then across all three tests (OFT, DLET, EPM). OFT (center entries), DLET (distance in the light chamber), and EPM (time in open arms) values were used to obtain emotionality *Z*-scores. Locomotion *Z*-scores were obtained from DLET (total distance traveled) and OFT (distance traveled in the border) data.

NOR. The NOR test was performed in a box measuring $l = 60$ cm, $w = 40$ cm, $h = 20$ cm. Mice were habituated to the arena for 5 min 24 h before the test. The test consisted of two sessions. In session 1, mice were allowed to explore two identical objects placed in the left and the right sides of the arena for 10 min. In session 2, 1 h after session 1, one of the familiar objects was changed with a novel object, and the mice were allowed to explore for 5 min. Objects were thoroughly cleaned between the sessions and between different mice. The process was video recorded, and the time that mice investigated (sniffed) the objects was measured manually (by an observer who was unaware of the treatment).

Preference for social novelty. The test was performed in the box measuring $l = 60$ cm, $w = 40$ cm, $h = 20$ cm that was separated into three equal compartments with clear-walled Plexiglas dividers with doors, allowing the mice to explore all three compartments freely. Each outer compartment also contained an inverted cup (Galaxy Pencil Cup/Utility Cup; Spectrum Diversified Designs, Inc., Streetsboro, OH). Before the beginning of the test, the mice were placed in the box for 5 min to habituate. The test consisted of two sessions. In session 1, an unfamiliar mouse was placed in the left inverted cup, and an object was placed in the right inverted cup. Then, the test mouse was free to explore all three compartments for 10 min. In session 2, the object in the right cup was replaced by a novel mouse. Again, the test mouse was free to explore all three compartments for 10 min. The process was video recorded, and the time that the test mouse investigated the object or the mice in the inverted cups were measured manually (by an observer who was unaware of the treatment).

Free social interaction. The social interaction test was performed in a clear Plexiglas box measuring $l = 30$ cm, $w = 30$ cm, $l = 20$ cm. Mice were first habituated to the arena for 5 min 24 h before the test. On the day of the test, the subject mice were paired with age- and sex-matched unfamiliar mice from the D2Cre/LSL-Flag-Cas9 colony for 10 min. The entire process was video recorded, and the time spent in social interaction (sniffing and grooming the partner) was measured manually (by an observer who was unaware of the treatment).

Supplementary References

S1. Sanz E, Yang L, Su T, et al. Cell-type-specific isolation of ribosome-associated mRNA from complex tissues. *Proc Natl Acad Sci U S A* 2009;106:13939–13944. DOI: 10.1073/pnas.0907143106.

S2. Reimand J, Isserlin R, Voisin V, et al. Pathway enrichment analysis and visualization of omics data using g:Profiler, GSEA, Cytoscape and EnrichmentMap. *Nat Protoc* 2019;14:482–517. DOI: 10.1038/s41596-018-0103-9.

S3. Khlghatyan J, Evstratova A, Chamberland S, et al. Mental illnesses-associated Fxr1 and its negative regulator Gsk3β are modulators of anxiety and glutamatergic neurotransmission. *Front Mol Neurosci* 2018;11:119. DOI: 10.3389/fnmol.2018.00119.

S4. Brinkman EK, Chen T, Amendola M, et al. Easy quantitative assessment of genome editing by sequence trace decomposition. *Nucleic Acids Res* 2014;42:e168. DOI: 10.1093/nar/gku936.

S5. Guilloux JP, Seney M, Edgar N, et al. Integrated behavioral z-scoring increases the sensitivity and reliability of behavioral phenotyping in mice: relevance to emotionality and sex. *J Neurosci Methods* 2011;197:21–31. DOI: 10.1016/j.jneumeth.2011.01.019.
